# Genomic analysis of a large set of currently—and historically—important human adenovirus pathogens

**DOI:** 10.1038/s41426-017-0004-y

**Published:** 2018-02-07

**Authors:** Ashrafali M. Ismail, Tiange Cui, Kalpana Dommaraju, Gurdeep Singh, Shoaleh Dehghan, Jason Seto, Susmita Shrivastava, Nadia B. Fedorova, Neha Gupta, Timothy B. Stockwell, Rebecca Halpin, Ramana Madupu, Albert Heim, Adriana E. Kajon, Eric G. Romanowski, Regis P. Kowalski, Jambulingam Malathi, Kuzhanthai L. Therese, Hajib Narahari Madhavan, Qiwei Zhang, Leonardo J. Ferreyra, Morris S. Jones, Jaya Rajaiya, David W. Dyer, James Chodosh, Donald Seto

**Affiliations:** 1 Department of Ophthalmology, Howe Laboratory, Massachusetts Eye and Ear Infirmary, Harvard Medical School, Boston, MA 02114 USA; 2 Bioinformatics and Computational Biology Program, School of Systems Biology, George Mason University, Manassas, VA 20110 USA; 3 Chemistry Department, American University, Washington, DC 20016 USA; 4 J. Craig Venter Institute, Rockville, MD 20850 USA; 5 Institut für Virologie, Medizinische Hochschule Hannover, Hannover, 30625 Germany; 6 Lovelace Respiratory Research Institute, Albuquerque, NM 87108 USA; 7 Charles T. Campbell Ophthalmic Microbiology Laboratory, Ear and Eye Institute, School of Medicine, University of Pittsburgh, Pittsburgh, PA 15213 USA; 8 L & T Microbiology Research Center, Kamalnayan Bajaj Research Center, Sankara Nethralaya, No. 41 College Road, Chennai, 600006 Tamil Nadu India; 9 Biosafety Level-3 Laboratory, School of Public Health, Southern Medical University, Guangzhou, 510515 China; 10 Institute of Virology “J. M. Vanella”, Enfermera Gordillo Gómez s/n, Agencia 4, Ciudad Universitaria, CP 5016 Córdoba, Argentina; 11 University of California Berkeley, School of Public Health, Berkeley, CA 94704 USA; 12 Department of Microbiology and Immunology, University of Oklahoma Health Sciences Center, Oklahoma City, OK 73104 USA

## Abstract

Human adenoviruses (HAdVs) are uniquely important “model organisms” as they have been used to elucidate fundamental biological processes, are recognized as complex pathogens, and are used as remedies for human health. As pathogens, HAdVs may effect asymptomatic or mild and severe symptomatic disease upon their infection of respiratory, ocular, gastrointestinal, and genitourinary systems. High-resolution genomic data have enhanced the understanding of HAdV epidemiology, with recombination recognized as an important and major pathway in the molecular evolution and genesis of emergent HAdV pathogens. To support this view and to actualize an algorithm for identifying, characterizing, and typing novel HAdVs, we determined the DNA sequence of 95 isolates from archives containing historically important pathogens and collections housing currently circulating strains to be sequenced. Of the 85 samples that were completely sequenced, 18 novel recombinants within species HAdV-B and D were identified. Two HAdV-D genomes were found to contain novel penton base and fiber genes with significant divergence from known molecular types. In this data set, we found additional isolates of HAdV-D53 and HAdV-D58, two novel genotypes recognized recently using genomics. This supports the thesis that novel HAdV genotypes are not limited to “one-time” appearances of the prototype but are of importance in HAdV epidemiology. These data underscore the significance of lateral genomic transfer in HAdV evolution and reinforce the potential public health impact of novel genotypes of HAdVs emerging in the population.

## Introduction

Human adenoviruses (HAdVs) occupy an important unique niche in biology and medicine as they were not only among the first respiratory viral pathogens to be isolated, identified, and characterized, but were also model organisms for fundamental discoveries and insights into molecular and cellular biology, immunology, and systems biology. These include messenger RNA splicing^1^, eukaryotic DNA replication^2^, and antigen presentation to T-cells^3^. Remarkably, this double-stranded (ds) DNA virus, with a capsid comprising nearly one-million amino acids and 150-megadalton molecular weight, has been crystallized and its structure resolved at 3.5 angstroms, allowing for insights into virus assembly and cell entry mechanisms^4^. This provides opportunities for improving and refining adenovirus-mediated gene transfer as vectors for vaccination and gene therapy^4,5^; and enhancing the role of HAdVs in oncolytics^6^.

As human pathogens, a wide spectrum of diseases is associated with HAdVs, involving the respiratory, ocular, gastrointestinal, and genitourinary systems, as well as a metabolic disorder (obesity)^7^. These have been documented extensively in the literature and many pathogenic strains have been collected into archives. Based on their biology, pathogenic attributes, and DNA sequence similarities, HAdVs are divided phylogenetically into seven species, A through G^7^. Adenovirus genotypes classified as HAdV-B, -C, and -E are associated principally with respiratory disease; HAdV-A, -D, -F, and -G with gastrointestinal disease; and HAdV-D and E with ocular diseases, including epidemic keratoconjunctivitis, a severe ocular surface infection^7^. HAdV-A genotypes have historical significance as oncogenic in certain rodent model organisms^8,9^. In immunocompetent individuals, HAdV infections are usually self-limiting and death is relatively uncommon^7^, but epidemics involving certain historically important strains and genotypes, e.g., “Ad-7h”, recently relabeled as type 66 (GenBank accession no. JN860676), “prove the exception”^10–12^. In immunocompromised individuals, HAdV infections are of significant concern, causing fatalities^7^. Specifically, HAdV-A, -B, and -C are all associated with infections of allogenic transplant recipients^7^, while many novel types within HAdV-D were first identified in patients with AIDS^13^ and other immunocompromised patients as potential opportunistic pathogens^14^. Thus, HAdV is a major public health concern in both immunocompetent and immunocompromised individuals.

As a model organism, adenoviruses are proving useful for demonstrating the power and application of high-resolution data from genomics and bioinformatics in pathogen detection^15^, analysis, and characterization. HAdVs are examples in which high-resolution sequence data are applied to resolve viral taxonomy^16^. Whole-genome sequencing and phylogenomics have now fully supplanted serology as the system by which HAdVs are typed^16,17^. To date, 84 unique HAdV genotypes (http://hadvwg.gmu.edu) are recognized by the adenovirus research community and by NCBI, with all genome-associated data deposited in GenBank. The availability of high-resolution genomic data has provided insights into the molecular evolution of this human viral pathogen. The genome of this presumably “stable” dsDNA virus, with respect to point mutations and genetic drift, is remarkably “unstable” as co-infections allow for homologous recombination to be a major pathway in the molecular evolution of new types and emergent pathogens^14,18–24^. This mechanism allows for “non-pathogenic” types to be “converted” into highly contagious pathogens^21,23^ and for a “renal pathogenic” type to be “converted” into a highly contagious respiratory pathogen^22^. Recent genomic analyses of thirty-two newly emergent adenoviral pathogens have demonstrated this mechanism in six HAdV species, with most reporting on the two HAdV species with the largest number of members, HAdV-B (11/84; 13.1%) and -D (54/84; 64.3%). Novel simian adenoviruses (SAdVs) also have been shown to arise via recombination^25^. These studies supplement the high-resolution re-analysis of the original 52 serotypes and confirm they are unique genotypes^19^, even among “major serological” cross-reacting types such as HAdV-D15 and -D29, which were controversial as they shared the identical serotyping epsilon antigen^26^.

In order to determine the degree to which previously untyped novel genotypes of HAdVs may be isolated from patients, two clinical centers evaluating ocular diseases were recruited (Sankara Nethralaya and University of Pittsburgh). Two collections of respiratory HAdV pathogens were included, providing a comparison of another disease system (Guangzhou and NHRC). To complement these, three archives containing historically important HAdV pathogens were included (Loveless Respiratory Research Institute, University of Florida, and Germany). The archives include serotyped but not genome-sequenced deposits, for example, “Ad-7h” which caused an unusual and atypical high rate of morbidity and mortality in the 1980 and 1990s^10–12^. Collectively, these efforts resulted in the first large-scale adenovirus study in which whole-genome data and DNA sequence analyzes were used to discriminate, type, and characterize HAdV pathogens. Two recently recognized HAdV genotypes, HAdV-D53 and -D58, were sampled serendipitously. These analyses confirm genomic recombination as a major molecular evolution mechanism in the genesis of novel HAdV pathogens and reaffirm HAdVs as on-going threats to public health, as well as provide an algorithm for rapid genomic sequencing and in silico identification and characterization of emergent viral pathogens.

## Materials and methods

### Sample selection and processing

Adenoviral samples were obtained from one researcher and six contemporary collections and archives. Selection criteria included sampling then-currently circulating pathogens of interest to the researchers and sampling “intriguing” historical isolates that were archived. The Germany archive included adenoviruses collected by two researchers (Drs. T. Adrian and R. Wigand) who have retired and have gifted the isolates to Dr. Albert Heim. From the Emerging Pathogens Institute (University of Florida; Gainesville, FL), a collection that was gathered from other researchers was made available. All adenoviruses were identified by serotyping methods by the original researchers and supplied as samples from eye, nasopharynx, pharynx, endotracheum, and feces, with the exception of the Germany archival samples and China samples, which were supplied as purified DNA, and the Florida archival samples, which were supplied as purified viruses. Samples were processed by several study centers: U.S. Naval Health Research Center collection (San Diego, CA) (processed at the Lovelace Respiratory Research Institute; Albuquerque, NM); University of Pittsburgh collection (Pittsburgh, PA) and Sankara Nethralaya collection (Chennai, India) (processed at the Massachusetts Eye and Ear Infirmary; Boston, MA); Biosafety Level-3 Laboratory, School of Public Health, Southern Medical University (Guangzhou, China); archives of Drs. T. Adrian and R. Wigand (Homburg, Germany) (processed at the Institut für Virologie, Medizinische Hochschule; Hannover, Germany); and one isolate from Dr. Leonardo Ferreyra (Cordoba, Argentina) (processed at the California Public Health Department; Richmond, CA). Additional historically important isolates were purchased from a NIH-funded archive at the Emerging Pathogens Institute of the University of Florida, Gainesville (Dr. Gary L. Heil). *N.B*., This collection is no longer available.

Samples were processed similarly at each collaborating laboratories^14,21,27,28^; for example, at the MEEI each virus was grown in either A549 cells, a human alveolar carcinoma cell line or HEp-2, a human laryngeal carcinoma cell line, and purified by CsCl gradient ultra-centrifugation. DNA was extracted either using a phenol–chloroform method or with a QIAmp MinElute Virus Spin Kit (Qiagen GmbH; Hilden, Germany). Most eye specimens were unpassaged and directly used for DNA extraction. The MEEI Human Studies Committee exempted this study from requiring informed consent as the samples were de-identified and would otherwise have been discarded.

### Genome sequencing

Purified genomic DNA was simultaneously PCR-amplified and bar-coded in two separate reactions using sequence independent single-primer amplification (SISPA) (Djikeng et al.^29^). All genomes were sequenced using a two-platform strategy: Ilumina HiSeq (San Diego, CA) and Roche 454 GS-FLX (Branford, CT). The SISPA products were normalized and pooled into a single sample that was purified using a PCR purification kit (Qiagen). This sample was subsequently gel purified to select for SISPA products that were 300–500 bp in size for Illumina HiSeq-based sequencing and 500–800 bp in size for 454 GS-FLX-based sequencing. In preparation for sequencing, two aliquots each were used to construct the 454 and Illumina libraries, and sequenced on the respective platforms. Following DNA sequencing, sequence reads from each platform were deconvoluted by barcode identity, and the sequences trimmed for quality and for removal of the SISPA hexamer primer sequences. All reads were then assembled de novo using “clc_novo_assembly”, a command-line assembly algorithm (https://www.qiagenbioinformatics.com/products/clc-genomics-workbench/) and the resulting contigs were BLAST-searched against a database of complete HAdV sequences available at GenBank to find the closest reference sequence. Both 454 GS-FLX and Illumina sequence reads were then mapped to the selected reference genome using the “clc_ref_assembly_long” command-line assembly algorithm. At loci where both 454 GS-FLX and Illumina sequence data agreed on a variation (as compared to the reference sequence), the reference sequence was updated to reflect the variation. A final mapping of all next-generation sequences to the updated reference sequences was performed with “clc_ref_assemble_long” command-line assembly algorithm. As most genomes showed significant variation compared to selected reference sequences, manual reference extension and editing was performed based on sequencing reads, followed by another round of mapping assembly as mentioned above. Furthermore, to improve resulting genome consensus and fill in sequencing gaps, custom primers were designed using the automated primer design software^30^, and targeted PCR-based DNA sequencing reactions were conducted. The PCR products were sequenced using Sanger dideoxy chemistry for short-range amplicons (up to 1 kb) or using IonTorrent sequencing platform (Thermo Fisher Scientific) for long-range amplicons (2–4 kb). These finishing reads were then merged with initial data using clc command-line assembly algorithm as mentioned above. The sequences were verified for functional completeness and adequate sequence coverage using in-house QA software tools (JCVI; Rockville, MD). Resultant finished genome sequences from this pipeline had average genome coverages of 214.2x.

### Preliminary high-throughput genome annotation and identification of penton base, hexon, and fiber genes

The sequences were annotated with a viral annotation software, Viral Genome ORF Reader (VIGOR)^31^, prior to GenBank submission. To identify and to query for novel penton base, hexon, and fiber genes, BLAST was used as well as a genotyping software tool for the hexon hypervariable regions, loops 1 and 2. This genotyping tool was developed by Kalpana Dommaraju (Ph.D. dissertation, in preparation) following the criteria published by Madisch et al.^32^. It also included typing of the penton base using the hypervariable region 1 and the RGD loop sequences and typing of the fiber by its knob sequence. Following visual inspection and editing, genome sequence and annotation data were deposited into the GenBank as part of Bioproject id PRJNA70469.

Following GenBank retrieval, additional higher resolution annotation of individual genomes of interest was performed using GATU (https://www.viprbrc.org) with manual methods to ensure accuracy. GenBank records will be updated to reflect these refined annotations.

### Computational genome analyses

Publicly accessible software tools were used to perform DNA sequence analyses, as described earlier^21^. Exceptions are noted below in the discussion of specific analyses. The NIH version of BLAST was used to query for sequences similarities against the GenBank database (http://blast.ncbi.nlm.nih.gov/Blast.cgi).

### Phylogenetic analysis

Sequences were aligned using the ClustalW tool in the Molecular Evolutionary Genetic Analysis version 6 software package (MEGA6; www.megasoftware.net/). Phylogenetic trees were constructed using the maximum-parsimony method with a bootstrap test of 1000 replicates and the Tree-Bisection-Reconnection (TBR) model. The whole-genome tree was drawn using the “Interactive Tree of Life” software (iTOL v3; itol.embl.de).

### Nucleotide diversity

Nucleotide diversity plots were constructed using the DNA Sequence Polymorphism software (DnaSP v5.10.01; www.ub.edu/dnasp/). Sites with alignment gaps were excluded. The analysis was performed with a sliding window length of 100 bps and step size of 25 bps. Pairwise sequence identities from multiple sequence alignments were calculated using the Sequence Identities and Similarities software (SIAS; http://imed.med.ucm.es/Tools/sias_help.html).

### Recombination analysis

Simplot, a web-accessible software tool, was used to query the genomes and genes for nucleotide sequence recombination. This software includes Bootscan, a tool that was used to complete the nucleotide sequence recombination assessment of the ClustalW-aligned genes^33^. For genes, default parameter settings were used for the window size (200 nucleotides [nt]), step size (20 nt), replicates used (*n* = 100), gap stripping (on), distance model (Kimura), and tree model (neighbor-joining). Similarly, whole genomes were analyzed, starting with an initial alignment using ClustalW and following with recombination analysis using Simplot and Bootscan. For this much larger DNA sequence, only the window size and step size were altered (1000 and 200, respectively), with the remainder of the default parameters unchanged.

### Homology modeling

A homology penton base protein model was built in the Swiss-Model ExPASy software (swissmodel.expasy.org) using the crystal structure of HAdV-C2 as the template (Protein Data Bank [PDB] code 1X9TA). UCSF Chimera v1.9 (www.cgl.ucsf.edu/chimera/) was used for visualization and root-mean-square deviation (RMSD) analysis.

### Statistical analysis

Nucleotide identity differences between HAdV (A–D) species were analyzed using Kruskal–Wallis test and the data represented as a boxplot. A *P*-value of < 0.05 was considered statistically significant. This analysis was performed using GraphPad Prism v6.0 (GraphPad Software; San Diego, CA).

## Results

### Large-scale genomic and bioinformatic analysis of 95 adenoviruses

Ninety-five HAdV genomes isolated from currently circulating and historically intriguing pathogens were sequenced, albeit ten genomes were only partially completed due to template quality and quantity. However, the partial genomic sequences allow identification of the “marker” genes for determining HAdV molecular types, in accordance with GenBank- and ICTV-accepted practices of metagenomics^17,34^, and provided valuable information. As shown in Table 1, virus samples, stocks, or purified genomic DNA were obtained from one researcher and six collections and archives, with selection criteria determined by the collaborator and included sampling then-currently circulating pathogens of interest and “intriguing” historical isolates from archives. All of the adenoviruses were identified by serotyping methods by the original researchers and supplied as samples from eye, nasopharynx, pharynx, endotracheum, and feces, or noted as “unknown”.

**Table 1:** Inventory of HAdV isolates

									Sequence identity		
Taxon	GenBank name	Serotype	Accession no.	Collection date	Location	Sample origin	Collection	Genome	Penton base	Hexon	Fiber
Species A	USA/UFL_Ad31/2005/31[P31H31F31]	Ad31	KF268119	2005	CO, USA	Fecal isolate	U Florida	HAdV-A31	HAdV-A31	HAdV-A31	HAdV-A31
Species B	DEU/Ad34/1985/34[P35H34F7]	Ad34	KF268328	1985	DEU	Unknown	Germany	Novel	HAdV-B35	HAdV-B34	HAdV-B7
	DEU/Ad3/1988/3[P16H3F16]	Ad3	KF268315	1988	DEU	Unknown	Germany	Novel	HAdV-B16	HAdV-B3	HAdV-B16
	USA/Ad21 + 16H16/1965/76[P21H21F16]	Ad21 + 16H16	KF633445	1965	TX, USA	Lung sample	Germany	Novel^a^	HAdV-B21	HAdV-B21	SAdV-B35
	USA/UFL_Ad3/2004/3[P3/H3/F3]	Ad3	KF268195	2004	MO, USA	Nasal wash	U Florida	HAdV-B3	HAdV-B3	HAdV-B3	HAdV-B3
	USA/UFL_Ad3a50/2007/3[P3H3F3]	Ad3a50	KF268133	2007	CT, USA	Nasal wash	U Florida	HAdV-B3	HAdV-B3	HAdV-B3	HAdV-B3
	USA/CL_45/1988/3[P3H7F3]	Ad3	KF268132	1988	PA, USA	Ocular isolate	U Pittsburgh	HAdV-B3	HAdV-B3	HAdV-B3	HAdV-B3
	USA/UFL_Ad3a17/2007/3[P3H3F3]	Ad3a17	KF268131	2007	CT, USA	Nasal wash	U Florida	HAdV-B3	HAdV-B3	HAdV-B3	HAdV-B3
	USA/CL_46/1988/3[P3H3F3]	Ad3	KF268128	1988	PA, USA	Ocular isolate	U Pittsburgh	HAdV-B3	HAdV-B3	HAdV-B3	HAdV-B3
	USA/UFL_Ad3a51/2007/3[P3H3F3]	Ad3a51	KF268123	2007	CT, USA	Nasal wash	U Florida	HAdV-B3	HAdV-B3	HAdV-B3	HAdV-B3
	USA/UFL_Ad3a2/2007/3[P3H3F3]	Ad3a2	KF268120	2007	CT, USA	Nasal wash	U Florida	HAdV-B3	HAdV-B3	HAdV-B3	HAdV-B3
	IND/Ad3/2011/3[P7H3F3]	Ad3	KF268212	2011	Tamil Nadu, INDIA	Ocular isolate	India	HAdV-B3	HAdV-B7	HAdV-B3	HAdV-B3
	IND/Ad3/2011/3[P7H3F3]	Ad3	KF268210	2011	Tamil Nadu, INDIA	Ocular isolate	India	HAdV-B3	HAdV-B7	HAdV-B3	HAdV-B3
	USA/Ad3/X/3[P3H3F3]	Ad3	KF268202	2003	PA, USA	Ocular isolate	U Pittsburgh	HAdV-B3	HAdV-B3	HAdV-B3	HAdV-B3
	USA/ak34_Ad3a2/2008/3[P3H3F3]	Ad3a2	JX423382	2008	Philadelphia, USA	Unknown	LRRI/NHRC	HAdV-B3	HAdV-B3	HAdV-B3	HAdV-B3
	USA/ak33_Ad3a/2003/3[P3H3F3]	Ad3a variant	JX423381	2008	Philadelphia, USA	Unknown	LRRI/NHRC	HAdV-B3	HAdV-B3	HAdV-B3	HAdV-B3
	USA/ak32_Ad3a/2004/3[P3H3F3]	Ad3a variant	JX423380	2004	San Diego, USA	Unknown	LRRI/NHRC	HAdV-B3	HAdV-B3	HAdV-B3	HAdV-B3
	USA/Ad3/1988/NEW[P3H3F7]	Ad3	KF429752	1988	PA, USA	Ocular isolate	U Pittsburgh	HAdV-B3	HAdV-B3	HAdV-B3	HAdV-B3
	CHN/Ad4/2007/NEW[P3H3F7]	Ad4	KF268311	2007	Guangzhou, CHINA	Throat swab	So Med Univ	HAdV-B3	HAdV-B3	HAdV-B3	HAdV-B3
	USA/UFL_Ad7d2-3/unknown/7[P7H7F7]	Ad7d2–4	KF268135	2002	IA, USA	Nasal wash	U Florida	HAdV-B7	HAdV-B7	HAdV-B7	HAdV-B7
	USA/Ad4/1988/7[P7H7F7]	Ad4	KF268134	1988	PA, USA	Ocular isolate	U Pittsburgh	HAdV-B7	HAdV-B7	HAdV-B7	HAdV-B7
	USA/Ad7a/1988/7[P7H7F7]	Ad7a	KF268125	1988	PA, USA	Ocular isolate	U Pittsburgh	HAdV-B7	HAdV-B7	HAdV-B7	HAdV-B7
	USA/UFL_Ad7d2-2/unknown/7[P7H7F7]	Ad7d2-1	KF268117	2001	IA, USA	Nasal wash	U Florida	HAdV-B7	HAdV-B7	HAdV-B7	HAdV-B7
	USA/Ad4/1988/7[P7H7F7]	Ad4	KF429748	1988	PA, USA	Ocular isolate	U Pittsburgh	HAdV-B7	HAdV-B7	HAdV-B7	HAdV-B7
	CHN/Ad7/2011/7[P7H7F7]	Ad7	KF268316	2011	Guangzhou, CHINA	Throat swab	So Med Univ	HAdV-B7	HAdV-B7	HAdV-B7	HAdV-B7
	CHN/Ad7/2011/7[P7H7F7]	Ad7	KF268314	2011	Dongguan, CHINA	Throat swab	So Med Univ	HAdV-B7	HAdV-B7	HAdV-B7	HAdV-B7
	USA/ak39_Ad7d2/1997/7[P7H7F7]	Ad7d2	JX423387	1997	IL, USA	Pharyngeal swab	LRRI/NHRC	HAdV-B7	HAdV-B7	HAdV-B7	HAdV-B7
	USA/ak40_Ad7b/1997/7[P7H7F7]	Ad7b	JX423388	1997	IL, USA	Unknown	LRRI/NHRC	HAdV-B7	HAdV-B7	HAdV-B7	HAdV-B7
	USA/ak35_Ad7d2/2006/7[P7H7F7]	Ad7d2	JX423383	2006	MO, USA	Pharyngeal swab	LRRI/NHRC	HAdV-B7	HAdV-B7	HAdV-B7	HAdV-B7
	USA/UFL_Ad11/2005/11[P11H11F11]	Ad11	KF268121	2005	WI, USA	Urine isolate	U Florida	HAdV-B11	HAdV-B11	HAdV-B11	HAdV-B11
	USA/UFL_Ad34/2005/34[P34/H34/F34]	Ad34	KF268196	2005	TX, USA	Nasal wash	U Florida	HAdV-B34	HAdV-B34	HAdV-B34	HAdV-B34
	USA/UFL_Ad35/2004/35[P35H35F35]	Ad35	KF268124	2004	IA, USA	Urine isolate	U Florida	HAdV-B35	HAdV-B35	HAdV-B35	HAdV-B35
	EGY/ak37_Ad11a/2001/55[P14H11F14]	Ad11a	JX423385	2001	EGY	Pharyngeal swab	LRRI/NHRC	HAdV-B55	HAdV-B14	HAdV-B11	HAdV-B14
	ARG/ak36_Ad11a/2005/55[P14H11F14]	Ad11a	JX423384	2001	ARG	Unknown	LRRI/NHRC	HAdV-B55	HAdV-B14	HAdV-B11	HAdV-B14
	USA/UFL_Ad7h/2005/66[P7H7F3]	Ad7h	KF268126	2005	CT, USA	Nasal wash	U Florida	HAdV-B66	HAdV-B7	HAdV-B7	HAdV-B3
	ARG/ak38_Ad7h/2003/7[P7H7F7]	Ad7h	JX423386	2003	ARG	Unknown	LRRI	HAdV-B66	HAdV-B7	HAdV-B7	HAdV-B3
Species C	ARG/Ad1/2000/1[P1H1F1]	Ad1	JX173078	2000	ARG	Unknown	LRRI	HAdV-C1	HAdV-C1	HAdV-C1	HAdV-C1
	USA/Ad1/2004/1[P1H1F1]	Ad1	JX173086	2004	USA	Unknown	LRRI/NHRC	HAdV-C1	HAdV-C1	HAdV-C1	HAdV-C1
	USA/Ad1/2003/1[P1H1F1]	Ad1	JX173085	2003	USA	Unknown	LRRI/NHRC	HAdV-C1	HAdV-C1	HAdV-C1	HAdV-C1
	USA/Ad1/2003/1[P1H1F1]	Ad1	JX173083	2003	USA	Unknown	LRRI/NHRC	HAdV-C1	HAdV-C1	HAdV-C1	HAdV-C1
	USA/Ad1/2003/1[P1H1F1]	Ad1	JX173082	2003	USA	Unknown	LRRI/NHRC	HAdV-C1	HAdV-C1	HAdV-C1	HAdV-C1
	EGY/Ad1/2001/1[P1H1F1]	Ad1	JX173080	2001	EGY	Unknown	LRRI/NHRC	HAdV-C1	HAdV-C1	HAdV-C1	HAdV-C1
	USA/UFL_Ad1/2005/1[P1H1F1]	Ad1	KF268331	2005	CO, USA	Nasal wash	U Florida	HAdV-C1	HAdV-C1	HAdV-C1	HAdV-C1
	USA/Ad1/1988/1[P1H1F1]	Ad1	KF429744	1988	PA, USA	Ocular isolate	U Pittsburgh	HAdV-C1	HAdV-C1	HAdV-C1	HAdV-C1
	USA/UFL_Ad2/2004/2[P2H2F2]	Ad2	KF268130	2004	NY, USA	Nasal wash	U Florida	HAdV-C2	HAdV-C2	HAdV-C2	HAdV-C2
	USA/Ad2/2003/2[P2H2F2]	Ad2	JX173084	2003	USA	Unknown	LRRI/NHRC	HAdV-C2	HAdV-C2	HAdV-C2	HAdV-C2
	EGY/Ad2/2001/2[P2H2F2]	Ad2	JX173081	2001	EGY	Unknown	LRRI/NHRC	HAdV-C2	HAdV-C2	HAdV-C2	HAdV-C2
	ARG/Ad2/2002/2[P2H2F2]	Ad2	JX173079	2002	ARG	Unknown	LRRI	HAdV-C2	HAdV-C2	HAdV-C2	HAdV-C2
	ARG/Ad2/2005/2[P2H2F2]	Ad2	JX173077	2005	ARG	Unknown	LRRI	HAdV-C2	HAdV-C2	HAdV-C2	HAdV-C2
	USA/Ad2/1992/2[P2H2F2]	Ad2	KF268310	1992	PA, USA	Ocular isolate	U Pittsburgh	HAdV-C2	HAdV-C2	HAdV-C2	HAdV-C2
	USA/UFL_Ad5/2008/5[P2/H5/F5]	Ad5	KF268199	2008	CT, USA	Nasopharyngeal asp.	U Florida	HAdV-C5	HAdV-C2	HAdV-C5	HAdV-C5
	USA/Ad2/1988/5[P5H5F5]	Ad2	KF268127	1988	PA, USA	Ocular isolate	U Pittsburgh	HAdV-C5	HAdV-C5	HAdV-C5	HAdV-C5
	USA/Ad8/1990/5[P5H5F5]	Ad8	KF429754	1990	PA, USA	Ocular isolate	U Pittsburgh	HAdV-C5	HAdV-C5	HAdV-C5	HAdV-C5
	USA/ak31_Ad6/2007/6[P6H6F6]	Ad6	JX423389	2007	Philadelphia, USA	Unknown	LRRI/NHRC	HAdV-C6	HAdV-C6	HAdV-C6	HAdV-C6
	USA/UFL_Ad6/2005/6[P6H6F6]	Ad6	KF268129	2005	CO, USA	Fecal isolate	U Florida	HAdV-C6	HAdV-C6	HAdV-C6	HAdV-C6
Species D	DEU/Ad9/1984/9[P67H9F15]	Ad9	KF268206	1984	DEU	Unknown	Germany	Novel	HAdV-D67	HAdV-D9	HAdV-D15
	DEU/Ad32/1988/32[P23H32F62]	Ad32	KF268327	1988	DEU	Unknown	Germany	Novel	HAdV-D23	HAdV-D32	HAdV-D62
	DEU/Ad28/1987/28[P67H28F60]	Ad28	KF268320	1987	DEU	Unknown	Germany	Novel	HAdV-D67	HAdV-D28	HAdV-D60
	DEU/Ad46/1986/46[P49H46F65]	Ad46	KF268332	1986	DEU	Unknown	Germany	Novel	HAdV-D49	HAdV-D46	HAdV-D9
	DEU/Ad46/1987/46[P9H46F39]	Ad46	KF268211	1987	DEU	Unknown	Germany	Novel	HAdV-D9	HAdV-D46	HAdV-D39
	DEU/Ad17/1986/17[P17H17F17]	Ad17	KF268330	1986	DEU	Unknown	Germany	Novel	HAdV-D48	HAdV-D17	HAdV-D30
	DEU/Ad37/1988/37[P67H37F45]	Ad37	KF268324	1988	DEU	Unknown	Germany	Novel	HAdV-D67	HAdV-D37	HAdV-D45
	DEU/Ad38/1984/38[P22H38F17]	Ad38	KF268312	1984	DEU	Unknown	Germany	Novel	HAdV-D42	HAdV-D38	HAdV-D30
	DEU/Ad33/1986/33[P62H33F17]	Ad33	KF268322	1986	DEU	Unknown	Germany	Novel	HAdV-D48	HAdV-D33	HAdV-D30
	DEU/Ad37/X/NEW[P37H37F17]	Ad37	KF268208	Unknown	DEU	Unknown	Germany	Novel	HAdV-D37	HAdV-D37	HAdV-D17
	DEU/Ad32/1986/NEW[P38H32F27]	Ad32	KF268325	1986	DEU	Unknown	Germany	Novel	HAdV-D38	HAdV-D32	HAdV-D27
	DEU/Ad37/1988/37[P28H37F38]	Ad37	KF268334	1988	DEU	Unknown	Germany	Novel	HAdV-D28	HAdV-D37	HAdV-D38
	DEU/Ad15/1982/NEW[P33H56F56]	Ad15	KF268201	1982	DEU	Unknown	Germany	Novel	HAdV-D33	HAdV-D15	HAdV-D9
	DEU/Ad71/1987/71[P9H20F67]	Ad9	KF268207	1987	DEU	Unknown	Germany	Novel^b^	HAdV-D9	HAdV-D20	Novel^b^
	DEU/Ad30/1985/30[P38H63F44]	Ad30	KF268335	1985	DEU	Unknown	Germany	Novel^c^	Novel^c^	HAdV-D30	Novel^c^
	USA/Ad8/1991/8[P8/H8/F53]	Ad8	KF268198	1991	PA, USA	Ocular isolate	U Pittsburgh	HAdV-D8	HAdV-D8	HAdV-D8	HAdV-D8
	USA/Ad8/1991/8[P8/H8F/F53]	Ad8	KF268118	1991	PA, USA	Ocular isolate	U Pittsburgh	HAdV-D8	HAdV-D8	HAdV-D8	HAdV-D8
	USA/Ad8/X/8[P8H8F8]	Ad8	KF268321	Unknown	PA, USA	Ocular isolate	U Pittsburgh	HAdV-D8	HAdV-D8	HAdV-D8	HAdV-D8
	USA/Ad8/1992/8[P8H8F8]	Ad8	KF429751	1992	PA, USA	Ocular isolate	U Pittsburgh	HAdV-D8	HAdV-D8	HAdV-D8	HAdV-D8
	USA/Ad8/X/8[P8H8F8]	Ad8	KF268205	Unknown	PA, USA	Unknown	U Pittsburgh	HAdV-D8	HAdV-D8	HAdV-D8	HAdV-D8
	DEU/Ad12/1988/15[P15H15F15]	Ad12	KF268204	1988	DEU	Unknown	Germany	HAdV-D15	HAdV-D15	HAdV-D15	HAdV-D15
	USA/Ad10-Ad19/1989/37[P37/H37/F37]	Ad10/Ad19	KF268122	1989	PA, USA	Ocular isolate	U Pittsburgh	HAdV-D37	HAdV-D37	HAdV-D37	HAdV-D37
	USA/Ad37/X/37[P37H37F37]	Ad37	KF268203	Unknown	PA, USA	Ocular isolate	U Pittsburgh	HAdV-D37	HAdV-D37	HAdV-D37	HAdV-D37
	USA/UFL_Ad22/2005/53[P53/H53/F53]	Ad22	KF268197	2005	CT, USA	Conjunctival swab	U Florida	HAdV-D53	HAdV-D37	HAdV-D22	HAdV-D8
	USA/Ad8/1992/56[P56/H56/F56]	Ad8	KF268333	1992	PA, USA	Ocular isolate	U Pittsburgh	HAdV-D56	HAdV-D9	HAdV-D15	HAdV-D9
	DEU/Ad15/1982/56[P56H56F56]	Ad15	KF268329	1982	DEU	Unknown	Germany	HAdV-D56	HAdV-D9	HAdV-D15	HAdV-D9
	USA/Ad4/1992/NEW[P26H56F56]	Ad4	KF268313	1992	PA, USA	Ocular isolate	U Pittsburgh	HAdV-D56	HAdV-D9	HAdV-D15	HAdV-D9
	USA/Ad56/X/56[P9H15F9]	Ad9	KF268209	Unknown	PA, USA	Ocular isolate	U Pittsburgh	HAdV-D56	HAdV-D9	HAdV-D15	HAdV-D9
	ARG/Ad58/2010/58[P58H58F58]	unknown	KF268319	2010	Cordoba, ARG	Fecal isolate	Ferreyra/CaPH	HAdV-D58	HAdV-D49	HAdV-D58	HAdV-D29
	USA/Ad64/X/64[P64H64F64]	Ad22	KF268213	Unknown	PA, USA	Ocular isolate	U Pittsburgh	HAdV-D64	HAdV-D22	HAdV-D19	HAdV-D37
	USA/Ad/1989/8[P8H8F8]	Ad8	KF429753	1989	PA, USA	Ocular isolate	U Pittsburgh	N/A- partial	HAdV-D8	HAdV-D8	HAdV-D8
	USA/Ad8/1992/8[P8H8F8]	Ad8	KF429749	1992	PA, USA	Ocular isolate	U Pittsburgh	N/A- partial	HAdV-D8	Unsequenced	HAdV-D8
	USA/Ad8/1991/8[P8H8F8]	Ad8	KF429746	1991	PA, USA	Ocular isolate	U Pittsburgh	N/A- partial	HAdV-D8	HAdV-D8	HAdV-D8
	USA/Ad8/1991/8[P8H8F8]	Ad8	KF429743	1991	PA, USA	Ocular isolate	U Pittsburgh	N/A- partial	HAdV-D8	HAdV-D8	HAdV-D8
	USA/Ad8/1992/NEW[P26H56F56]	Ad8	KF429747	1992	PA, USA	Ocular isolate	U Pittsburgh	N/A- partial	HAdV-D9	HAdV-D15	HAdV-D9
	USA/Ad8/1992/NEW[P26H56F56]	Ad8	KF429750	1992	PA, USA	Ocular isolate	U Pittsburgh	N/A- partial	HAdV-D9	HAdV-D15	Unsequenced
	USA/UFL_Ad19/2005/64[P64H64F64]	Ad19	KF268323	2005	TN, USA	Bronchoscopy sample	U Florida	N/A- partial	HAdV-D22	HAdV-D19	HAdV-D37
	USA/UFL_Ad45/2005/[P-NEW/H45/F-NEW]	Ad45	KF268200	2005	IN, USA	Fecal isolate	U Florida	N/A- partial	HAdV-D48	HAdV-D45	Novel
	DEU/Ad17/1988/17[P29H17F30]	Ad17	KF268326	1988	DEU	Unknown	Germany	N/A- partial	HAdV-D29	HAdV-D17	HAdV-D29
	USA/Ad8/1992/NEW[P9H22F44]	Ad8	KF429745	1992	PA, USA	Ocular isolate	U Pittsburgh	N/A- partial	HAdV-D9	HAdV-D22	Unsequenced

*ARG* Argentina, *DEU* Germany, *EGY* Egypt. GenBank accession number, serotype, sample origin, collection information, and preliminary sequence characterization of 85 currently circulating and historically intriguing human adenoviruses (*HAdVs*) are presented. Complete and partial genome sequences were analyzed with respect to sequence percent identities for genotyping using the three major capsid genes: penton base, hexon, and fiber. Sequence identities to prototypes are noted, with the HAdV name noted as species and type. Novel genome and gene sequences are noted as “novel”, with three genomes re-named as accepted genotypes (1, HAdV-B76; 2, HAdV-D71; and 3, HAdV-D72). The current GenBank name is included for reference; these will be updated with regards to the sequence analyses. Collections sampled include the School of Public Health, Southern Medical University, Guangzhou, China (So Med Univ); Sankara Nethralaya, Chennai, India (India); Institut für Virologie, Medizinische Hochschule, Hannover, Germany (Germany); (U Florida); Charles T. Campbell Ophthalmic Microbiology Laboratory, Ear and Eye Institute, School of Medicine, University of Pittsburgh, Pittsburgh, PA (U Pittsburgh); and two collections processed at the Lovelace Respiratory Research Institute, Albuquerque, NM, including samples from Dr. Adriana Kajon and the U.S. Naval Health Research Center, San Diego, CA (LRRI and LRRI/NHRC, respectively). One sample was provided by Dr. Leonardo Ferreyra (Cordoba, Argentina) and processed at the California Public Health Dept., Richmond, CA (Ferreyra/CaPH).

^a^HAdV-B76

^b^HAdV-D71

^c^HAdV-D72

### Whole-genome analysis of sequenced HAdVs species (A–D)

Initial post-sequencing, JCVI pipeline-based, automated annotation, supplemented with two HAdV genotyping tools (in *beta* testing), provided a “first-pass” characterization of these genomes. These were submitted to GenBank, ahead of publication, and assigned accession numbers, as shown in Table 1.

**Fig. 1:**
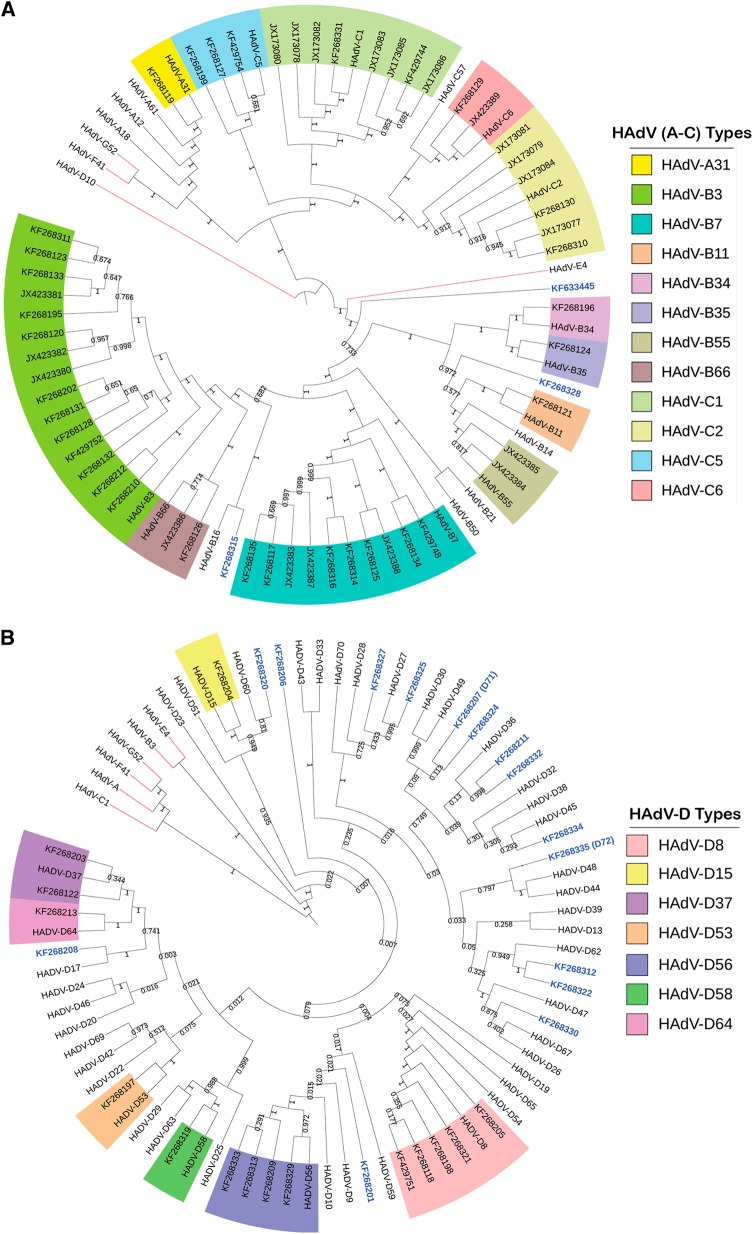
Human adenovirus whole-genome phylogenetic trees for **(a)** HAdV-A through C and **(b)** HAdV-D complete genomic sequences Two trees are presented in order to display the larger number of species D clearly. Representatives of species E, F, and G are presented for reference. Trees were constructed using the maximum-parsimony analysis, following alignment using the ClustalW tool in the Molecular Evolutionary Genetic Analysis version 6 software package (MEGA6; www.megasoftware.net/), with a bootstrap test of 1000 replicates and default parameters and Tree-Bisection-Reconnection (TBR) method. Each clade is highlighted according to specific HAdV species using a color code as shown. Novel sequences are shown in blue and displayed as GenBank accession numbers (acc. nos.). Bootstrap values are displayed on the branches and nodes for different HAdV species used for references are shown in red

To provide a more thorough and individualized analysis of each whole-genome sequence, additional computational analyses were performed. The newly obtained sequences were typed to four HAdV species, including species A (*n* = 1), species B (*n* = 35), species C (*n* = 19), and species D (*n* = 40). Upon BLAST and whole-genome phylogenetic analysis, 67 sequences were characterized as previously known genotypes, as shown in the whole-genome phylogenetic trees (Fig. 1a, b). In these trees, the newly obtained genomes are noted by their GenBank accession numbers; there are two trees presented in order to highlight the larger HAdV-D sample size. Representative genomes from species E, F, and G are included for reference. The remaining 18 sequences showed 1.65 to 7.48% nucleotide differences and diverged into separate subclades from existing HAdV types. These sequences were considered as novel genotypes, and were identified as either HAdV-B or D. One novel genome, KF633445, clades with species B and appears to have a zoonotic origin (data not shown).

By maximum-parsimony phylogenetic analysis, one sequence in HAdV-A grouped with type 31, an important pathogen in allotransplant recipients^7^. Within the HAdV-B genotypes, the majority of isolates segregated with types B3 (*n* = 15), B7 (*n* = 11), and B55 (*n* = 2). This reflected the collections and respiratory pathogen interests of two of the collaborators, and comprised the majority of samples in the University of Florida archive of historically intriguing pathogens. The genotype distribution in species HAdV-C were C1 (*n* = 7), C2 (*n* = 7), C5 (*n* = 3), and C6 (*n* = 2), again reflecting the above respiratory disease interests. For two other collaborators, ocular disease-associated pathogens are represented by the majority of submitted samples; these genotypes are in species HAdV-D (43%) and segregate with isolates associated with epidemic keratoconjunctivitis, including HAdV-D8 (*n* = 5), -D37 (*n* = 2), -D53 (*n* = 1), -D56 (*n* = 4), and -D64 (*n* = 1), reflecting the clinical settings in which the samples were obtained. This multi-center, large-scale genomics study identifies a wide distribution of HAdV-A. B, C, and D genotypes. The genomes identified are consistent with those noted as respiratory and ocular pathogens in the literature, and were available given the interests of individual centers in these diseases, for example, HAdV-B7 with respiratory diseases and HAdV-D8 with ocular diseases.

### Sequence diversity of HAdVs genotypes in species A through D

**Fig. 2:**
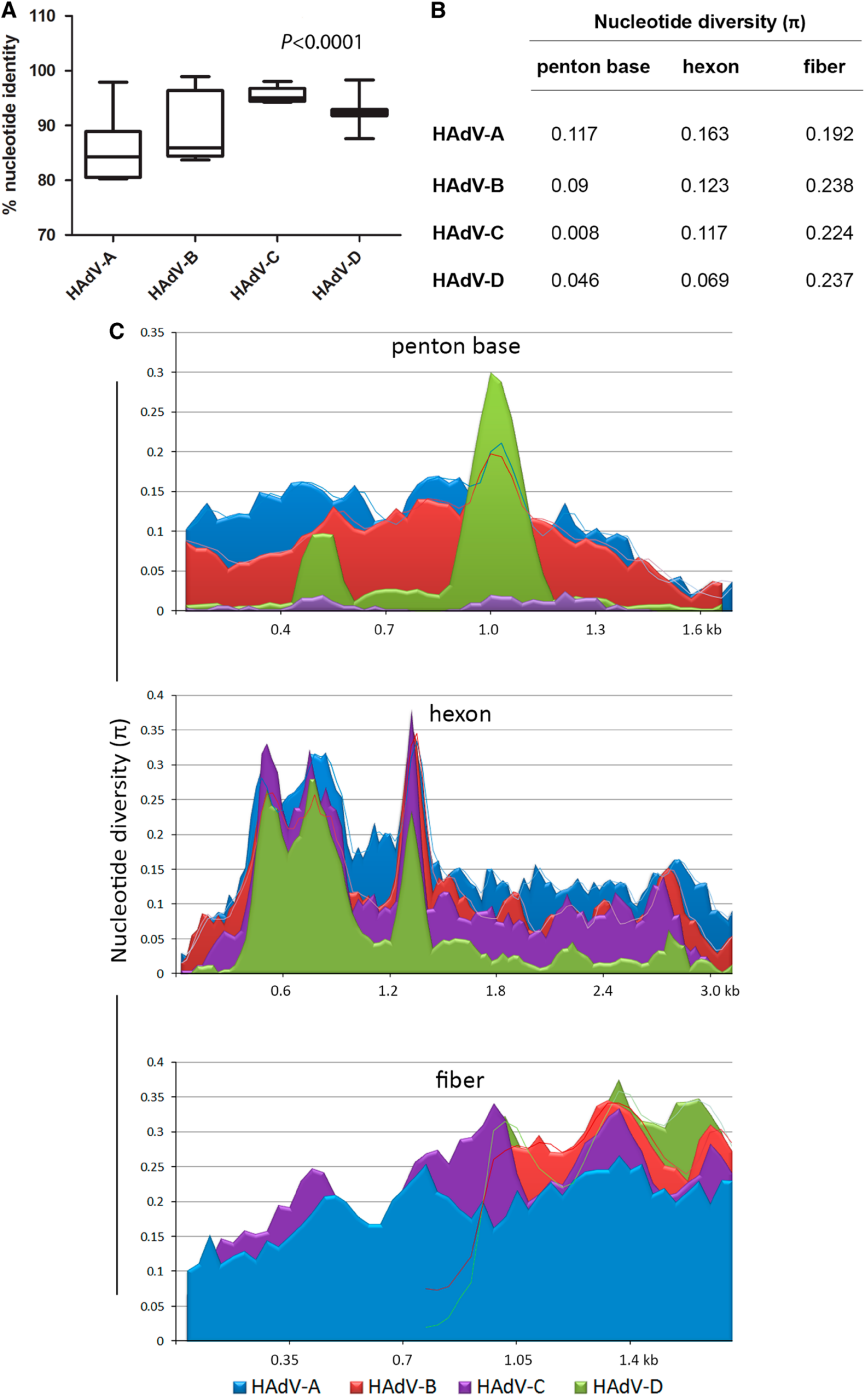
Nucleotide sequence identity and diversity data for HAdV species (A–D) prototype sequences **a** “Box-and-whiskers” plots show percent nucleotide identity of prototype HAdV complete genome sequences. The “box” represents the median and interquartile range (IQR), and the “whiskers” show both minimum and maximum values. The spacing between the boxes indicates the degree of spread; as depicted, the HAdV-C and D species are relatively homogenous. The nucleotide identities are significantly different for HAdV species analyzed (Kruskal–Wallis, *P* < 0.0001). **b** Average nucleotide diversity of the major capsid genes between each type within HAdV-A through D. **c** Nucleotide diversity (π) plots showing the average number of nucleotide differences per site along each gene for HAdV-A through D, calculated for penton base, hexon, and fiber prototype sequences. The plot was constructed using DnaSP v5 ((http://www.ub.edu/dnasp/), with a 100 nucleotide window and 25 nucleotide step size. Nucleotide alignments with gaps were excluded and graphs were constructed using Microsoft Excel software. The lines in the graph represent “trend lines”. Viruses within HADV-B and -D had shorter fiber genes than HAdV-A or -C and the trend lines are denoted accordingly

HAdV genome sequences are largely conserved from isolate to isolate, of the same type, e.g., HAdV-B7, over time^35–37^, as expected for dsDNA genomes. An analysis that included all of the prototype genome sequences for HAdV-A, -B, -C, and -D genotypes shows the average nucleotide identities within species were 85.5, 89.7, 95.5, and 92.3%, respectively (Fig. 2a). It was reconfirmed with this large data set that the genomic regions that primarily influence sequence and genome diversity include the three major capsid genes: penton base, hexon, and fiber, along with the E3 transcription unit (at distinct loci). These capsid genes are the same regions employed for HAdV genotyping and identification of recombinants^16,17^.

Nucleotide sequence diversity analyses and graphs comparing the major capsid genes for each type in HAdV-A through -D reaffirm the relationships reported in the literature (Fig. 2b, c). The nucleotide diversity plot (Fig. 2c) depicts the HAdV-C penton base genes to be relatively conserved, reflecting previous observations for these species C genotypes^38^. For HAdV-D, there are two distinct hypervariable regions (HVR-1 and 2) within the penton base gene; these correspond to the two hypervariable loop domains (HVL1 and HVL2) on the protein. In contrast, the penton base gene of HAdV-A and -B are entirely hypervariable. This is reflected also in average intra-species sequence divergence rates for the HAdV-A, -B, -C, and -D penton base gene of 11.7, 9.0, 0.8, and 4.6%, respectively (Fig. 2b).

The hexon protein contains two hypervariable loop domains (HVL1 and HVL2) that form the “epsilon” epitope, determinants that are recognized by neutralizing antibodies and are the basis for serum or virus neutralization (SN or VN) and serotyping^7,37,39^. For this data set, the average nucleotide divergence rates for the hexon gene was 16.3, 12.3, 11.7, and 6.9% for HAdV-A, -B, -C, and -D, respectively.

The trimeric fiber protein contains a N-terminal tail, a central shaft, and a C-terminal knob; the latter mediates the primary interaction with host cells, i.e., cell tropism. The fiber knob contains the “gamma” epitope, which was useful for serotyping through hemagglutination inhibition (HI)^7^. In comparison to the other major capsid genes, the fiber was found to be entirely hypervariable in all four HAdV species (Fig. 2c). Notably, the fiber open-reading frames for HAdV-B and D are just over half the length of those for HAdV-A and C, consistent with the literature, but all four species show a similar degree of diversity (Fig. 2b, c).

### Characterization of hypervariable capsid genes

**Fig. 3:**
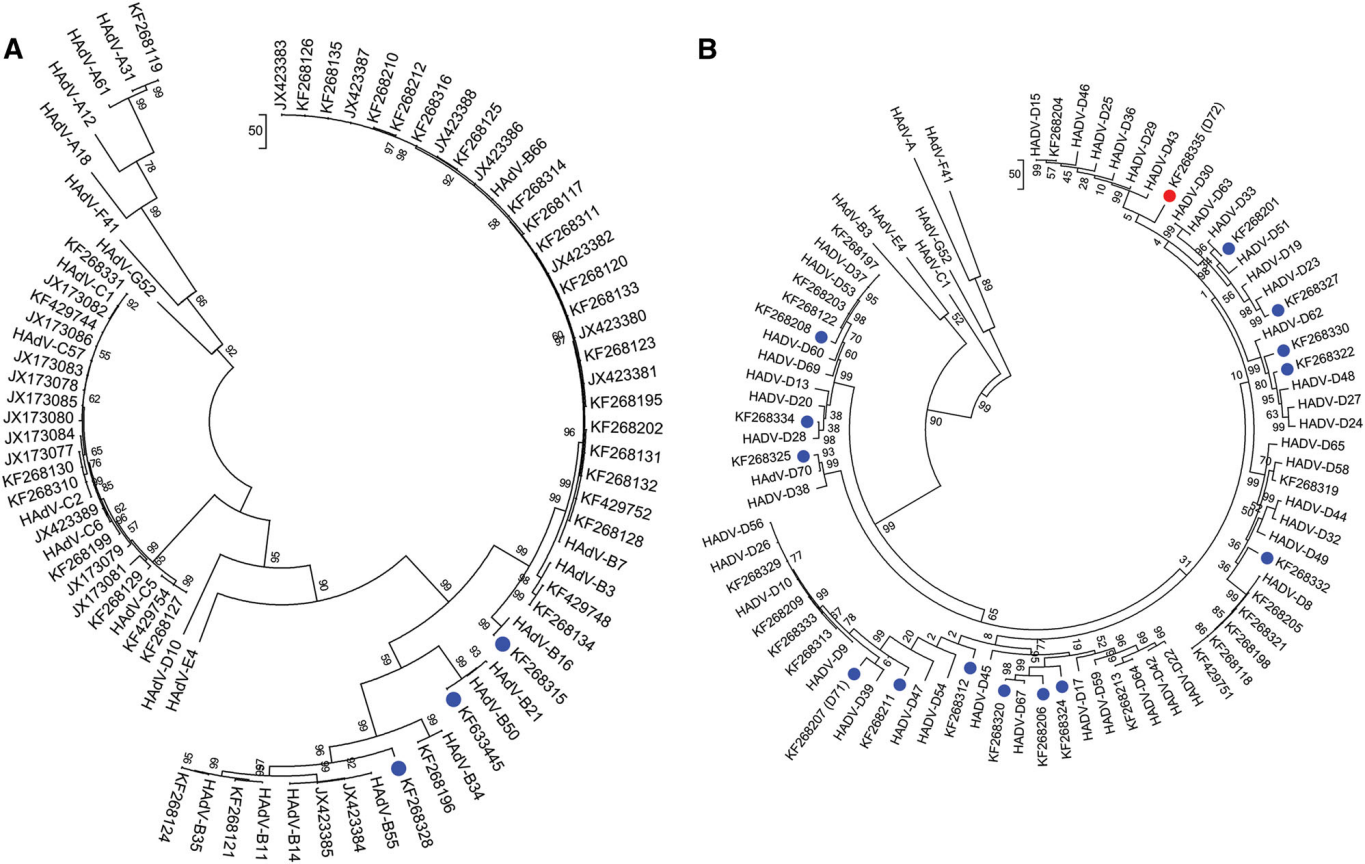
Maximum-parsimony phylogenetic analysis of the penton base Penton base gene sequences of **a** HAdV species A–C genotypes and **b** HAdV species D genotypes are presented; two trees allow for discrimination of the larger numbers of species D genotypes. Representatives of species E, F, and G are included for reference. Sequences obtained from this study are displayed as GenBank accession numbers. Novel HAdVs in this data set, identified by whole-genome analysis, are marked with blue dots. A novel penton base gene (KF268355; HAdV-D72) that diverged significantly from previously described HAdV-D sequences is identified by a red dot. Trees were constructed using the maximum-parsimony analysis, following alignment using the ClustalW tool in the Molecular Evolutionary Genetic Analysis v6 software package (MEGA6;www.megasoftware.net/), with a bootstrap test of 1000 replicates and default parameters, and Tree-Bisection-Reconnection (TBR) method in MEGA 6.0

**Fig. 4:**
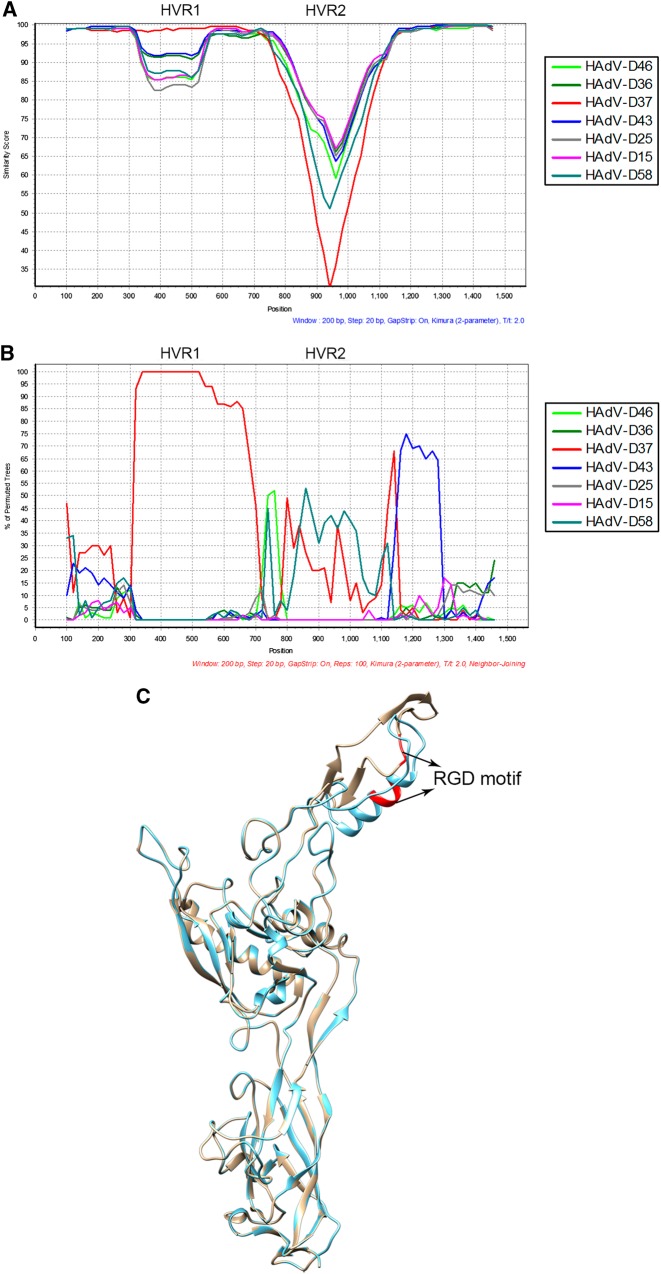
Recombination and structural modeling analysis of putative novel penton base gene in HAdV-D72 **a** SimPlot analysis demonstrates the genetic distances to HAdV reference sequences across the penton base gene, in which the *x*-axis denotes gene nucleotide position and the percentages of permutated trees that supported grouping are marked along the *y*-axis (http://sray.med.som.jhmi.edu/SCRoftware/simplot/). **b** Bootscan analysis demonstrates phylogenetic relationships to the reference strains. Each HAdV reference genotype is color coded; only closely related HAdV types are presented for clarity. Penton base gene recombination between hypervariable region (HVR)-1 and the RGD motif-containing HVR-2 is shown. The HVR-1 fragment showed high similarity with HAdV-D37, while HVR-2 was dissimilar to existing reference sequences, indicating both recombination and a novel gene segment. Prior to recombination analysis, sequences were aligned using the ClustalW tool in the Molecular Evolutionary Genetic Analysis v6 software package (MEGA6; www.megasoftware.net/). Default parameter settings for the Simplot software were used for analyzing the hexon sequences: window size (200 nucleotides [nt]), step size (20 nt), replicates used (n1/4100), gap stripping (on), distance model (Kimura) and tree model (neighbor-joining). **c** Homology modeling of the penton base in which the HAdV-D72 amino-acid sequences (blue) is superimposed over that of HAdV-D37 (cyan). Superimposition of structural models showed significant structural variation in RGD motif location (indicated in red). Homology model was built in Swiss ExPASy (http://swissmodel.expasy.org) using the crystal structure of HAdV-C2 as the template ([PDB] code 1X9TA)

To classify the molecular types for each adenoviral genome and to characterize in detail the 18 novel genotype sequences identified by whole-genome analysis (Fig. 1), maximum-parsimony phylogenetic analyses were performed for the capsid genes. The penton base phylogenetic tree for HAdV-A through -C and, separately, for HAdV-D are displayed in Fig. 3a, b, respectively. All three novel sequences in HAdV-B clustered to one of the previously known types B16, B35, and B21, with 99% bootstrap support (Fig. 3a). Among the 15 novel sequences in HAdV-D, 14 are clustered with the previously known types, with the majority showing 80–99% bootstrap support (blue dots, Fig. 3b). HAdV-D72 formed a separate subclade and is recognized as a novel genotype (Fig. 3b). Using Simplot and Bootscan software for sequence recombination analyses, the penton base sequence of HAdV-D72 was found to be recombinant, with its HVR-1 deriving from HAdV-D37 and its HVR-2 comprising a novel sequence (18% nucleotide divergence from nearest HAdV-D15), as shown in Fig. 4a, b, respectively. Penton base HVR-2 contains the canonical Arg-Gly-Asp (RGD) motif that interacts with host cell integrins to mediate virus endocytosis^40,41^. To examine this closer, a penton base homology protein model for this novel sequence was generated, superimposed with HAdV-D37 for visualizing the structural variations (Fig. 4c). The RMSD for the two superimposed structures was 0.419 with structural distance 8.54 and *Q*-value 0.869. In the structural model, crucial β-sheets for the novel RGD loop are absent and an additional α-helix is projected. Intriguingly, the RGD motif falls within the crucial α-helix. Novelties in the penton base and fiber (noted below) genes led to the approval by the Human Adenovirus Working Group and NCBI of a new type number (HAdV-D72) (Accession no. KF268335).

**Fig. 5:**
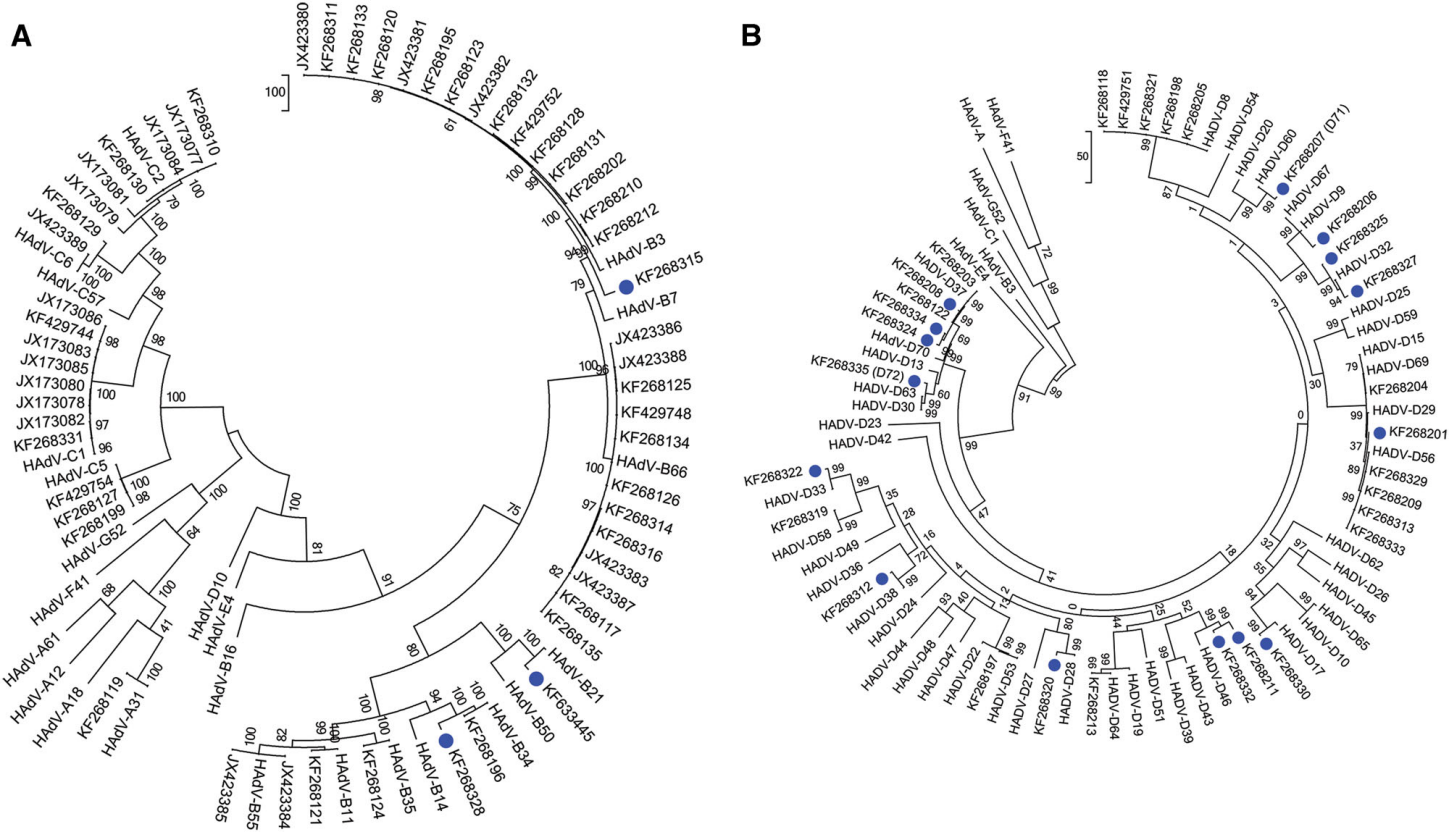
Maximum-parsimony phylogenetic analysis for hexon Gene sequences of hexons from **a** HAdV-A, -B, and -C and **b** HAdV-D are presented. Trees were constructed, following sequence alignment with ClustalW, using the maximum-parsimony option of the Molecular Evolutionary Genetic Analysis v6 software package (MEGA6; www.megasoftware.net/) and implementing a bootstrap test of 1000 replicates and default parameters. Sequences derived from this large-scale sequencing study are identified by their GenBank accession codes. Putative novel genotypes are denoted by blue dots. No novel hexon sequence was found

The hexon gene sequences were analyzed by maximum-parsimony phylogenetic analysis, with trees for HAdV-A, -B, and -C and for HAdV-D, separately, presented in Fig. 5a, b, respectively. Three novel HAdV-B genotypes contain hexon sequences that clustered with previously typed HAdV-B3, -B34, and -B21. Similarly, all HAdV-D hexon genes clustered with previously known types. Therefore, no unique hexon molecular types were identified in the whole and partial genome sequences of this data set.

**Fig. 6:**
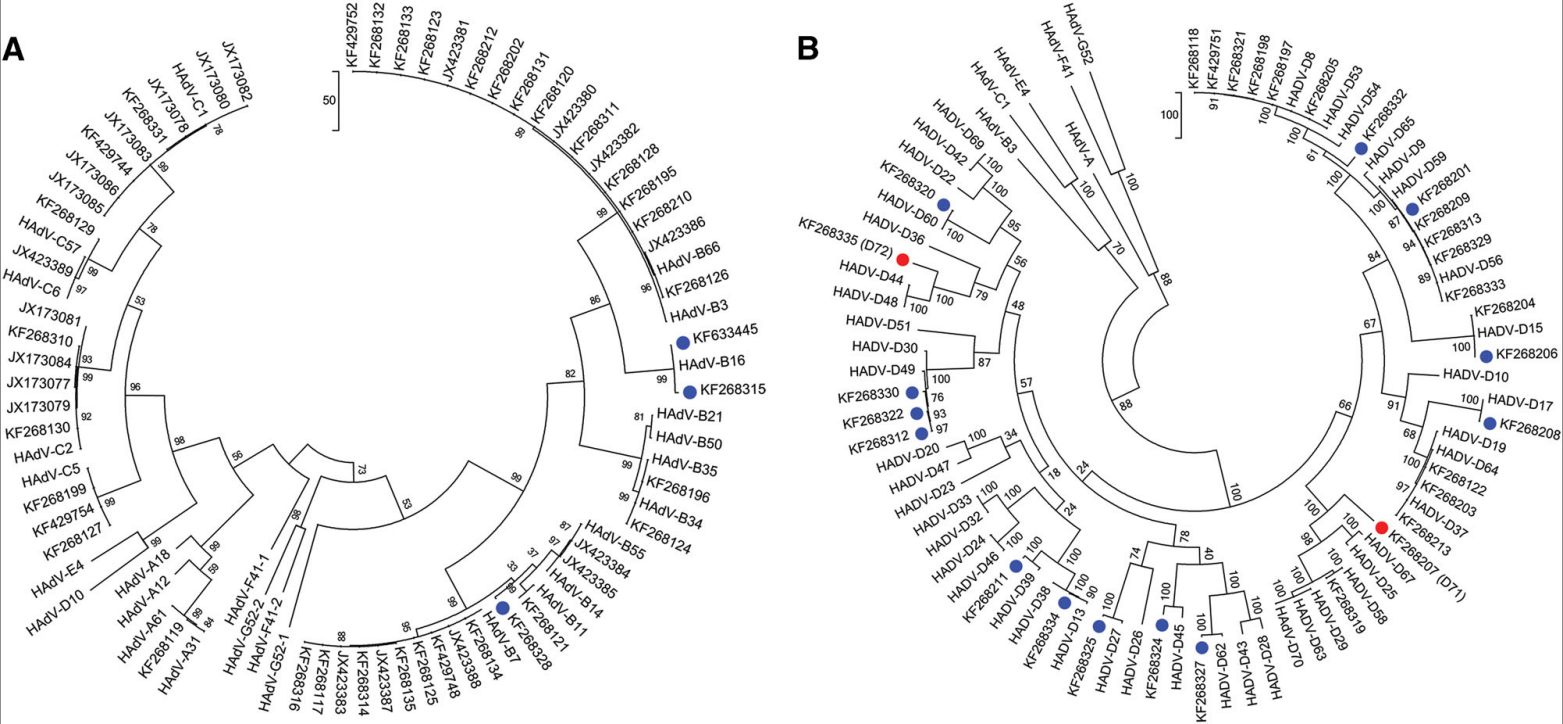
Maximum-parsimony phylogenetic analysis for the fiber genes Sequences of **a** HAdV-A, -B, and -C and **b** HAdV-D are presented as phylogenetic trees. Sequences derived from this large-scale sequencing study are identified by their GenBank accession codes. Novel HAdV genotype sequences identified by whole-genome analysis are denoted with blue dots. Novel fiber genes (KF268207, HAdV-D71, and KF268355, D72) with sequences that diverged significantly with known HAdV-D type sequences are identified by red dots. Trees were constructed, following sequence alignment with ClustalW, using the maximum-parsimony option of the Molecular Evolutionary Genetic Analysis v6 software package (MEGA6; www.megasoftware.net/) and implementing a bootstrap test of 1000 replicates and default parameters

Phylogenetic trees for the fiber genes of viruses sequenced in species HAdV-A, -B, and -C and for HAdV-D, separately, are shown in Fig. 6a, b, respectively. As displayed, the three novel HAdV-B genotypes contain fiber sequences that clustered with the HAdV-B7 (one) and -B16 fiber genes (two). Among the 15 novel sequences in HAdV-D, 13 contained fiber genes with homology to published counterpart sequences. The remaining two sequences each formed distinct clades with significant bootstrap support (red dots, Fig. 6b). Calculated nucleotide sequence differences to the nearest fiber sequences (HAdV-D67 and -D44) were 16% and 9%, respectively. These two emergent adenoviruses, with putatively novel fiber genes, were recognized with new type numbers by NCBI as HAdV-D71 and -D72 (KF268207 and KF268335, respectively). It is noteworthy that while HAdV-D72 contains both a novel penton base protein and novel fiber protein, it has a hexon gene sequence that is highly similar to HAdV-D30.

### HAdV molecular types and novel genotypes

**Fig. 7:**
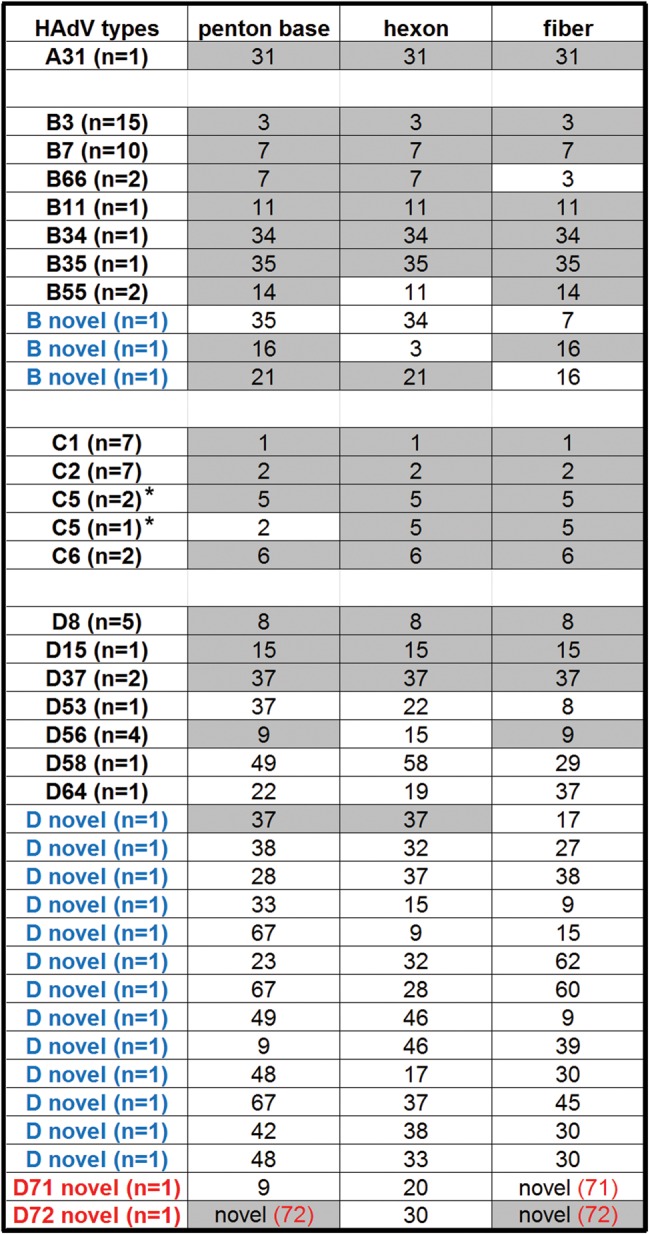
Summary of genotypes and molecular types For each isolate sequenced and providing whole-genome sequence, their capsid genes are noted, with the number of related isolates in parenthesis as “*n* = ”. Capsid genes that were shared within each HAdV type are highlighted. HAdV genotypes that are novel recombinants are indicated in blue and HAdV types containing novel genes indicated in red; novel genotype number is indicated in red and in parenthesis. *The penton bases for HAdV-C species are highly conserved and, therefore, these penton base genes did not contribute to HAdV typing in this species by whole-genome analysis

Eighty-five newly obtained whole-genome sequences isolated from respiratory, ocular, and gastrointestinal pathogens partition into four HAdV species: A (*n* = 1), B (*n* = 35), C (*n* = 19), and D (*n* = 40). The numbers are arbitrarily skewed due to the number of samples provided by collaborators and the success of the sequencing run due to the quality and quantity of the DNA. These genomes and their genotype-defining markers, i.e., the three major capsid genes, are presented in Fig. 7. It is clear that recombination is a major evolution pathway by which novel and emergent HAdV pathogens arise. Even with a skewed and “small” sample size of 85, recombination is observed in significant numbers. Of the 30 HAdV-D whole-genomes sequenced, 22 (73.3%) were clearly recombinants; for HAdV-B and -C, 07/35 (20%) and 01/19 (5.3%), respectively, were clear recombinants. In summary, 18 novel genotypes were found.

## Discussion

The Human Adenovirus (HAdV) Genome Sequencing Project was a collaborative project between the Adenovirus Genome Sequencing Consortium and the J. Venter Institute (JCVI) Genomic Sequencing Center for Infectious Diseases (GSC) (Rockville, MD). Using HAdV as a model organism, several sequencing platforms, sequencing strategies, and software tools were tested, modified, and/or developed. After test runs, the optimal small-genome sequencing strategy was a two-platform protocol: Ilumina HiSeq (San Diego, CA) and Roche 454 GS-FLX (Branford, CT). The advantages of each provided for the shortfalls inherent with the other. The protocol is designed to identify and type rapidly an unknown HAdV pathogen.

Whole-genome sequencing and preliminary bioinformatics analysis of 95 samples were performed to characterize the predominance of genotypes under certain conditions and to study the molecular evolution of HAdV pathogens, e.g., the numbers of recombinant viruses. Of the 85 whole-genomes sequenced, 18 are of previously uncharacterized HAdVs, all of which evolved by homologous recombination, including three novel HAdV-B types and 15 novel HAdV-D types. Partially sequenced viral genomes are hallmarks of metagenomics survey projects and have been accepted by both NCBI and ICTV recently^34^. In this survey, there are ten partial genome sequences, all of which were from samples collected in eye clinics. The large-scale sampling and processing, including variable viral titers, likely contributed to the genomic DNA quality and quantity. Even with partial sequences, three ocular isolates were characterized as highly similar to HAdV-D8, a known EKC pathogen, with penton base, hexon, and fiber sequences (P8H8F8) (KF429753, KF429746, and KF429743). For KF429749, the penton and fiber are also highly similar to HADV-D8, however, the hexon sequence is incomplete. KF429747 and KF429750 are both recombinants at the three capsid genes used for HAdV typing, having sequence similarities corresponding to a penton base of HAdV-D9 and a hexon of HAdV-D15. The latter has an incomplete fiber gene sequence and the former has a fiber sequence with identity to HAdV-D9. KF268323 is similar to HAdV-D64 with a preliminary recombinant genotyping of P22H19F37. Similarly, KF268326 is similar to HAdV-D17, with a preliminary genotyping as P29D17F29. KF268200 has a preliminary genotype of P48H45 with a novel fiber. The final partial genome KF429745 was noted with a preliminary genotype of P9H22 and an unsequenced fiber gene.

In the continuation of understanding sequence divergence and proteotypes^19^, two novel fiber genes and one novel penton base gene were defined in two viruses within species HAdV-D. These analyses confirm genome recombination and lateral genomic transfer as a major molecular evolution mechanism in the genesis of novel HAdV pathogens and provide an algorithm for rapid genomic sequencing and in silico identification and characterization of emergent viral pathogens. Genomics and bioinformatics allow a thorough and high-resolution analysis of currently circulating pathogens, in some cases, providing insights into why a particular isolate is either more or less pathogenic, infectious, or contagious^42^. This approach is invaluable to understanding pathogens that were similarly historically intriguing but no longer circulating. As an example, a respiratory adenoviral pathogen causing a fatality and isolated in the U.S. was studied and archived in a Germany repository. It was identified as an adenovirus that serotyped at the epsilon epitope as both HAdV-B21 and -B16, both acute respiratory disease (ARD) pathogens, and at the gamma epitope as HAdV-B16. It was named “Ad21 + 16H16”, which was not standard. Following its genome determination in this study, subsequent analysis beyond serotyping the hexon and fiber reveals it was an emergent and is a predicted “coming” human pathogen. Uniquely, it contains a multi-recombinant genome that incorporates elements of two SAdVs along with two HAdVs, suggesting multi-directional and reciprocal zoonosis and anthroponosis (prototype (HQ883276 preparation).

Another goal was to assist NCBI/GenBank in standardizing the HAdV genome records by providing an example to the research community, in providing a large set of similarly annotated and formatted HAdV genome data, including an informative GenBank “universal” name that included “adenovirus species/host/location/lab name/year/type number [serological/genome markers]”; and example of this is “Adenovirus D human/DEU/IAI-1/2005/53[P37H22F8].”

This is the first large-scale adenovirus study in which whole-genome data and DNA sequence analyses were used to discriminate and to type and characterize HAdV pathogens. In the past, only serology-based typing of the epsilon (SN) and/or gamma (HI) epitopes were reported. The gamma epitope is notoriously difficult to determine let alone repeat, even by “experts”^26,43^. Given the numbers of recombinants, the past surveys are flawed for a deeper understanding of this pathogen. For a comprehensive understanding of what makes a pathogen a pathogen, and to what degree, a solid definition of what exactly constitutes a pathogen is critical. Three examples illustrate this clearly. A novel recombinant strain of HAdV-D22, (“22H8”) with a penton sequence identical to type 37 was reported as a highly contagious epidemic keratoconjunctivitis (EKC) pathogen in Germany^44^. Since its first isolation in 1960 and to date, HAdV-D22 had never been associated with EKC^45^. Genome analysis showed the emergent pathogen was a recombinant that incorporated only the epsilon epitope of HAdV-D22 into the genome chassis that contained a penton based derived from HAdV-D37 and a fiber transferred from HAdV-D8, both of which are EKC pathogens^21^; it was named HAdV-D53 in recognition of a novel adenoviral pathogen. Furthermore, in a subsequent re-analysis of a collection of EKC pathogens at the Japan National Institute of Infectious Diseases, based on data from Walsh et al.^21^, HAdV-D53 was found to have been a major circulating ocular pathogen since 1996 and was the third most common EKC pathogen, having been mis-identified “as types 8, 22, or 37” (ref.^46^). The correct identification focuses efforts to prevent or remedy HAdV-D37-caused EKC infections to be directed at HAdV-37 chassis rather than the non-pathogenic HAdV-D22. A similar example was reported for the emergent EKC pathogen and recombinant HAdV-D64, comprising the epsilon epitope of non-pathogenic HAdV-D19 and the EKC pathogen HAdV-D37^23^. A third example is that of the “Trojan Horse” pathogen HAdV-B55^22^. This is a highly contagious ARD pathogen that is a recombinant that presents the epsilon epitope of a renal pathogen, HAdV-B11 in a genome and proteome of an ARD pathogen, HAdV-B14. No other HAdV-B11 strain or genome types are respiratory pathogens. HAdV-B11 has been associated with renal disease in immunocompromised renal transplant patients^7^ and likely has limited circulation in the general population, hence an immunologically naive population as HAdV-B14 circulates currently^47–49^.

This study provides support that the novel recombinant genotypes are relevant pathogens despite possible sporadic reports subsequent to the initial identification and recognition of the prototype. As noted earlier for HAdV-D53, recognition of a novel pathogen may be followed by re-examination of archives and subsequent identification of mistyped pathogens^46^. Interestingly, HAdV-D53 was found in an archive of historically intriguing pathogens, collected in Connecticut (USA; 2005). It was typed serologically and mis-identified as HAdV-D22, a non-EKC pathogen of low virulence. The genome data (KF268197) presented in this study shows identity to the prototype HAdV-D53, and reinforces the importance of high-resolution data in identifying and typing pathogens for a better understanding of their molecular evolution, distribution, and epidemiology. This recognition of a third sampling and third country of isolation indicates that HAdV-D53 and other novel HAdV genotypes may be underreported and underappreciated as important human pathogens, particularly if only the hexon and/or fiber serotyping epitopes are used for identity and typing.

Additionally, a specimen associated with gastrointestinal disease was included. It was isolated from a stool sample of an AIDS patient (aged 46) in 1996 and was initially typed by hexon sequencing as HAdV-F41. The virus isolated and amplified in cell culture was thought to be interesting as it was not serum neutralized by antiserum to HAdV-F41. However, upon genome sequencing, this isolate shows sequence identity to prototype HAdV-D58 (HQ883276), isolated in 1996 from the stool of a 31-year-old AIDS patient who presented with severe chronic diarrhea. Interestingly, HAdV-D58 (KF268319) contained a serologically unique hexon and a recombinant fiber that has a partial proximal shaft sequence derived from HAdV-D25 and a distal shaft plus knob contributed by HAdV-D29. A comparison of the sequences of KF268319 to the prototype reveals a nearly identical virus: Genome (99.4%); penton base (99.3%); hexon (99.8%); and fiber (100%). In retrospect, the patient, with chronic diarrhea, had a co-infection of two HAdVs; apparently, HAdV-F41 did not replicate well in cell culture prior to DNA isolation for sequencing. It should be noted that one objection to using genome data for recognizing novel HAdV types, as opposed to only hexon and fiber epitopes, was that many “newly-recognized” HAdV species D serotypes from AIDS patients in the 1990s were never reported again. In the context of HAdV evolution and pathogen genesis, this objection is irrelevant, as demonstrated for this isolate of HAdV-D58 and for the previously noted HAdV-D53.

Viruses represent a highly tractable model system for studying evolutionary biology. The HAdV genome is largely conserved, but interrupted in stereotypical fashion by hypervariability at four major regions of the genome, specifically the three major capsid genes and the E3 transcription unit. By comparison of genomic differences between HAdV major capsid regions, marked differences across species were noted. For example, the penton base genes of HADV-C types are relatively conserved. In contrast, HAdV-A and -B penton base genes are largely hypervariable across their entire open-reading frames. HAdV-D penton base genes show overall conservation interrupted by two distinct hypervariable regions, accounting for two distinct hypervariable loops on the external capsid surface, HVL1 and HVL2. We previously showed that these two hypervariable regions of the penton base gene often undergo homologous recombination, and at times, independent of one another^50^. HAdV-D72 was typed as a novel genotype on the basis of differential recombination within the penton base gene, specifically involving RGD loop (HVL2). Thus, it appears that viruses within HAdV-D may more frequently utilize penton base gene recombination for their evolution than other HAdV species. Also notable was the fiber gene. Viruses within HADV-B and -D had shorter fiber genes than HAdV-A or -C, but for all the four species, the fiber sequences showed a similar degree of diversity. A short trimeric fiber protein is thought to be more rigid, perhaps with more strict receptor interactions and cellular specificity. However, a short fiber may permit docking of penton base proteins to host cell integrins independent of fiber knob binding to a primary adenovirus receptor, and allow for fiber-independent cell entry^51^. These data suggest that fiber length matters, but in a complex fashion. Furthermore, we and others have shown a correlation between fiber knob amino acids and corneal tropism^42,52^. Altogether, these data are consistent with an important role for variations in fiber length and nucleotide content in infectivity and cellular tropism.

The range of HAdV types in circulation reflects an indeterminate combination of asymptomatic carrier states and the global burden of disease caused by adenoviruses, and we lack sufficient information about the human source of each sample and their condition to draw conclusions. However, HAdV-A31, the only HAdV-A identified in this study, is a pathogen of allogeneic hematopoietic stem cell transplant recipients. In contrast, a majority of HAdV-B types identified were -B7 and -B3, both associated with severe respiratory infections, along with the HAdV-B55 discussed above. HAdV-Cs are important pathogens in immunocompromised persons and are primarily associated with respiratory infection. Infections from viruses within HAdV-C are all commonly seen in young children. In our analysis, all the HAdV-Cs we identified had been previously characterized.

Identification of two HAdV-D types with novel fiber genes suggests as yet unidentified parent viruses for which the full genomes have not yet been characterized. Though the RGD loop was not solved in the original crystal structure (1X9T), our modeling analysis also suggested significant structural variation in the penton base protein RGD loop of the aforementioned HAdV-D72. The RGD motif is thought to be crucial to adenovirus internalization, and the RGD motif predicted within a novel α-helix structure in HAdV-D72 likely has an impact on cellular interactions. Though the conformation of the protein in this disordered loop is not informative, identification of α-helix over β-sheets in the comparative modeling analysis could be significant. This further warrants the need for deeper high-resolution structural analysis. Notably, the fiber protein of HAdV-D72 was also novel.

Altogether, HAdV types characterized in this survey were all from the four HAdV species with the most members, and were either replicates of viruses already whole-genome sequenced and characterized or newly identified recombinants, and two of the latter contained novel penton base and/or fiber genes. All HAdV-Ds with archived whole-genome sequences show evidence for homologous recombination in at least two loci in their genomes. In our study, recombination among HAdV-Ds was much more common than finding novel capsid genes, supporting the primacy of homologous recombination in the molecular evolution of HAdV-D. Our data further support evolution of HAdV-B by homologous recombination, but less assuredly of HAdV-C. However, the numbers of total viruses within HAdV-C was too few to allow for a conclusion. Finally, the clinical significance of those novel HAdV types identified in this analysis remains unknown. Regardless, our results taken together with recent descriptions of putatively new HAdV types causing serious diseases, highlights HAdV evolution as a persistent threat to public health.
