# Author Correction: Genomic analysis of a large set of currently—and historically—important human adenovirus pathogens

**DOI:** 10.1038/s41426-018-0200-4

**Published:** 2018-12-08

**Authors:** Ashrafali M. Ismail, Tiange Cui, Kalpana Dommaraju, Gurdeep Singh, Shoaleh Dehghan, Jason Seto, Susmita Shrivastava, Nadia B. Fedorova, Neha Gupta, Timothy B. Stockwell, Rebecca Halpin, Ramana Madupu, Albert Heim, Adriana E. Kajon, Eric G. Romanowski, Regis P. Kowalski, Jambulingam Malathi, Kuzhanthai L. Therese, Hajib Narahari Madhavan, Qiwei Zhang, Leonardo J. Ferreyra, Morris S. Jones, Jaya Rajaiya, David W. Dyer, James Chodosh, Donald Seto

**Affiliations:** 1 Department of Ophthalmology, Howe Laboratory, Massachusetts Eye and Ear Infirmary, Harvard Medical School, Boston, MA 02114 USA; 2 Bioinformatics and Computational Biology Program, School of Systems Biology, George Mason University, Manassas, VA 20110 USA; 3 Chemistry Department, American University, Washington, DC 20016 USA; 4 J. Craig Venter Institute, Rockville, MD 20850 USA; 5 Institut für Virologie, Medizinische Hochschule Hannover, Hannover, 30625 Germany; 6 Lovelace Respiratory Research Institute, Albuquerque, NM 87108 USA; 7 Charles T. Campbell Ophthalmic Microbiology Laboratory, Ear and Eye Institute, School of Medicine, University of Pittsburgh, Pittsburgh, PA 15213 USA; 8 L &, T Microbiology Research Center, Kamalnayan Bajaj Research Center, Sankara Nethralaya, No. 41 College Road, Chennai, Tamil Nadu 600006 India; 9 Biosafety Level-3 Laboratory, School of Public Health, Southern Medical University, 510515 Guangzhou, China; 10 Institute of Virology “J. M. Vanella”, Enfermera Gordillo Gómez s/n, Agencia 4, Ciudad Universitaria, CP 5016 Córdoba, Argentina; 11 School of Public Health, University of California Berkeley, Berkeley, CA 94704 USA; 12 Department of Microbiology and Immunology, University of Oklahoma Health Sciences Center, Oklahoma City, OK 73104 USA

Correction to: Emerging Microbes & Infections (2018)**7**:10 10.1038/s41426-017-0004-y

The original publication of this Article [1] was missing the below author that made contributions to the research and the published Article:

Rebecca Halpin

During the submission process, the name of Halpin was unintentionally omitted. In this correction article, the revised author list is provided and the original publication has been updated to state the contribution of Halpin.
